# Passively mode-locked fiber laser by a cell-type WS_2_ nanosheets saturable absorber

**DOI:** 10.1038/srep12587

**Published:** 2015-07-27

**Authors:** Peiguang Yan, Aijiang Liu, Yushan Chen, JinZhang Wang, Shuangchen Ruan, Hao Chen, Jinfei Ding

**Affiliations:** 1 Shenzhen key laboratory of laser engineering, Key Laboratory of Advanced Optical Precision Manufacturing Technology of Guangdong Higher Education Institutes, College of Optoelectronic Engineering, Shenzhen University, Shenzhen, 518060, China

## Abstract

A cell-type saturable absorber has been demonstrated by filling the single mode photonic crystal fiber (SMPCF) with tungsten disulfide (WS_2_) nanosheets. The modulation depth, saturable intensity, and non-saturable loss of this SA are measured to be 3.53%, 159 MW/cm^2^ and 23.2%, respectively. Based on this SA, a passively mode-locked EDF laser has been achieved with pulse duration of 808 fs and repetition rate of 19.57 MHz, and signal-noise-ratio (SNR) of 60.5 dB. Our results demonstrate that the cell-type WS_2_ nanosheets SA can serve as a good candidate for short-pulse mode locker.

Ultrafast fiber lasers have many widespread applications in optical communication, industrial material processing, optical sensing and biomedical diagnostics^1^,^2^,^3^. Compared with the active mode-locking technique^4^, passive mode-locking technique has the advantages of compactness, simplicity and flexibility. In the past research, nonlinear polarization rotation (NPR)^5^ and semiconductor saturable absorber mirror (SESAM)^6^ are two commonly used techniques because they provide fast amplitude modulation. However, NPR suffers from bulky construction and environmental sensitivity. SESAM requires complicated fabrication and packaging process, and has limited bandwidth (typically few-tens nm [6]). In 2004, carbon nanotube (CNT) has been widely employed in passively mode-locked fiber lasers as saturable absorber (SA) owing to its distinguished properties, *e.g.* ultrafast recovery time, ease of fabrication and integration into fiber cavity^7^,^8^,^9^,^10^. Since then, the high-performance SA interest was migrated onto the low-dimension nanomaterials due to their remarkable optical and electrical properties. Graphene has an intrinsic property of broadband operation arising from its gapless linear dispersion of Dirac electrons, making it different from the CNT with diameter-dependent operation band^11^,^12^,^13^,^14^,^15^,^16^,^17^,^18^,^19^,^20^,^21^,^22^,^23^. Thus, graphene has boosted the tremendous research activity in SAs for passive mode-locking of lasers in recent years. But graphene also holds two main disadvantages, the weak modulation depth (typically ~1.3% per layer) and the difficulty of creating an optical bandgap. Hence, significant efforts are currently being directed towards the study of other 2D nanomaterials beyond graphene^24^, such as topological insulators (TIs)^25^,^26^,^27^,^28^,^29^,^30^,^31^,^32^,^33^,^34^,^35^, transition mental dichalcogenides (TMDs) including of molybdenum disulfide (MoS_2_)^36^,^37^,^38^,^39^,^40^,^41^,^42^,^43^,^44^,^45^ or tungsten disulfide (WS_2_)^46^,^47^,^48^ as well as their diselenide analogues^49^, and black phosphorus^50^,^51^. By manipulating the thickness or atomic defects, the bandgap of TMDs can be engineered^44^. TMDs, such as MoS_2_, have been reported with broadband saturable absorption^36^, high third-order nonlinear susceptibility^37^ and ultrafast carrier dynamics^38^.

In laser cavity, the nanomaterials are usually hosted into polymeric film and pasted on fiber ferule, or deposited on microfiber and side polished fiber (SPF). However, because that the light directly pass through the polymeric film with low softening point, the damage threshold is relatively low for the polymeric SA. Although the damage threshold is evidently enhanced for the microfiber-type^41^ and SPF-type SAs^32^,^33^, the main problems are listed as follows: 1) low repeatability, the deposition of nanomaterial on microfiber and SPF is not precisely controllable; 2) the frangibility as a device, both microfiber and SPF are harmed to access the evanescent wave around fiber core; 3) Unwanted polarization-dependent loss (PDL) by the drawing or polishing of fiber. In other approach, photonic crystal fiber (PCF)^52^ can play as an optical platform by filling the air channels with various nanomaterials to form functional devices for mode-locking^53^,^54^,^55^,^56^. Owing to adjacence of core region, the nanomaterial can be effectively penetrated into by the evanescent wave around the surface of fiber core. As a result, the light-matter interaction is quite strong along the filled length. In 2011, Z. B. Liu first reported a nanosecond-pulse erbium-doped fiber (EDF) laser that was passively mode locked by a hollow-core PCF filled with few-layered graphene oxide solution. To data, 2D materials, such as graphene and layered TIs, have been embedded into PCFs and employed as SAs in ultrafast photonics. Although PCF-based SA can overcome the above problems in microfiber- or SPF-based SAs, this type of SA device also has the following problems: 1) Relatively larger insertion loss (IL), which might arise from the lower splicing efficiency between SMF and PCF, also from the PCF segment filled with nanomaterials. For PCF-based SA, too long a PCF segment might introduce too large absorption or loss into the cavity, which can make the laser too difficult to self-start; 2) Distortion of the guiding mode in the PCF region, which usually introduces the Mach-Zehnder interferometer (MZI) effect that is unfavorable for the stable mode-locking.

In this paper, we propose a cell-type of SA by filling WS_2_ nanosheets into a single-mode PCF (SMPCF) with length of 90 μm. The SMPCF-based cell-type SA can lower the IL and suppress the MZI effect by reducing PCF length and intermode interference. The modulation depth, saturable intensity and non-saturable loss are measured to be 3.53%, 159 MW/cm^2^ and 23.2%, respectively. Based on this SA, a passively mode-locked EDF laser has been achieved with pulse duration of 808 fs and repetition rate of 19.57 MHz, and signal-noise-ratio (SNR) of 60.5 dB. Our results demonstrate that the cell-type WS_2_ nanosheets SA can serve as a good candidate for short-pulse mode locker.

The WS_2_ nanosheets solution is commercially available from the Xfnano.com, which is prepared by dispersing 26 mg of WS_2_ nanosheets into 1 L of ethanol. Figure 1(a,b) shows the surface and thickness of the as-prepared WS_2_ nanosheets measured by scanning electron microscope (SEM), respectively. It can be seen that the nanosheets exhibit the layered structure appearance with the width of ~500 nm and the thickness of ~23.1 nm. It should be pointed out that the nanosheets are nonuniform. The width and length are in the range from 50 nm to 500 nm, and the thicknesses are from monolayer to ~25 nm, provided by the Xfnano.com.

For conveniently measuring the linear absorption, Raman spectrum, the solution was dripped onto a silica glass substrate, and then evaporated to dryness in an oven. We measured the linear-absorption spectrum from 250 to 2500 nm using an optical spectrometer (Perkinelmer Lambda 7500). The linear-absorption curve is shown in Fig. 2(a), which is smooth in infrared region with 4.9% absorption at 1.56 μm, indicating the potential of the few-layer WS_2_ as a broadband optical material. The dip at 630 nm on the transmission spectrum could be attributed to the direct gap transition, which proves the existence of monolayer WS_2_ in as-prepared sample^57^,^58^. Figure 2(b) shows the measured Raman spectrum of WS_2_ nanosheets using excitation of 514 nm. It has the typical Raman peaks, *e.g.* two optical phonon modes (E_2g_^1^ at 355.7 cm^−1^ and A_1g_ at 419.7 cm^−1^) and two typical longitudinal acouaaaastic modes (LA(M) at 175 cm^−1^ and 351 cm^−1^, where the E_2g_^1^ is an in-plane optical mode and A_1g_ corresponds to the out-of-plane vibrations along the c-axis direction of the S atoms. It is remarkable that the intensity of the strongest A_1g_ mode at 419.7 cm^−1^ is higher than the intensity of the E_2g_^1^ mode at 355.7 cm^−1^, which is different from that of monolayer WS_2_^59^.

The PCF, provided by YOFC.com, has a core/cladding diameter of 9.63/120 μm, as shown in Fig. 3(a). The core size is close with that of SMF-28 fiber, which can reduce their splicing loss. The air channels in PCF have average diameter/pitch of 3.05/6.4 μm, corresponding to an air fraction of 47.7%. Figure 3(b) shows the calculated mode distribution. It should be pointed out that the PCF sustain single mode operation in telecommunication band. Once it is used as SA platform in laser cavity, the distortion of guiding mode can be greatly suppressed. In order to fabricate a cell-type SA, three steps are carried out. In the 1^st^ step, the PCF is filled with WS_2_ nanosheets solution by high-pressure injection method. In this process, we first cut the end face of the PCF by an optical fiber cleaver, and then put this end of the PCF into the injector with WS_2_ nanosheets solution. The AB glue is used to wrap their intersection location tightly to avoid the leakage of solution between the PCF and the injector; In the 2^nd^ step, the solution-filled PCF is oven dried at a fixed temperature of 60 degree for 3 hours to remove the ethanol solvent and leave only the WS_2_ nanosheets in the air channels. In the 3^rd^ step, we use a multiple-cutting method to get the PCF-based cell-type SA. We first splice two SMF-28 fibers at the both sides of a short piece of nanosheets-filled PCF, and then cut the PCF part by a fiber cleaver. Subsequently, we select the part of shorter remained PCF and splice its end with the SMF-28 fiber. With repeatedly cutting and splicing, the remained length of PCF is only 90 μm, near to the thickness of common polymeric-composites SAs^46^. Figure 3(c,d) shows the side-view image of the single splicing point and SA device, respectively. The total IL of this cell-type SA device is measured to be ~1.5 dB at 1560 nm. This loss level here is lower than that of 4 dB in ref. [56] and above 6 dB in ref. [55]. Because the guiding mode is symmetrical, the SA doesn’t exhibit evident PDL effect. Considering the arcing harm on the nanosheets in PCF channels during splicing, it is not necessary to further shorten its length.

The linear transmission of the SA device is measured in the range from 1250 nm to 1600 nm by using an ASE source (Glight, 1250 nm ~ 1650 nm) and optical spectrum analyzer (OSA). The transmittance at 1560 nm is 73%. The fluctuations in spectrum show the existence of interference effect. But this effect is negligible beyond 1500 nm, which does not force the laser to operate with multiple wavelengths, thus disturbing the mode-locking stability. To investigate the nonlinear saturable absorption property of the as-prepared SA device, the standard 2-arm transmission measurement scheme is carried out. A home-made femtosecond laser (central wavelength: 1562 nm, repetition rate: ~22.5 MHz, pulse duration: 650 fs) is utilized as test source, a variable optical attenuator (VOA) is applied to continuously change the input optical intensity into the sample. A 50:50 optical coupler (OC) is used to split the laser into two arms with one arm for power-dependent transmission measurement of SA device and the other arm for reference. As increasing the optical intensity from 30 MW/cm^2^ to 500 MW/cm^2^ into the SA device, the results are recorded and depicted in Fig. 4(b). The results show that the transmittance of SA is increased by about 3.53% (Δα: modulation depth) to a level of 76.8%. The saturable intensity (I_sat_) is at a level of 159 MW/cm^2^, and non-saturable loss (α_ns_) is about 23.2%.

Recent works have reported the saturable absorption parameters of few-layer WS_2_ nanosheets for different type of Sas, e.g. Δα of 2.96%, I_sat_ of 362 MW/cm^2^ and α_ns_ of 30.9% for polymeric-composites-film SA in ref. [46]; Δα of 1.8% and I_sat_ of 750 MW/cm^2^ for polymeric-composites-film SA in ref. [47]; Δα of 0.95% and I_sat_ of 600 MW/cm^2^ for SPF-based SA. Our SA has a lower I_sat_ and higher Δα compared with the SPF-based SA in ref. [47]. In our SA, the WS_2_ nanosheets just adhere in the air channels surrounding the PCF core region, leading to a stronger light-matter interaction than SPF-based SA. The nanosheets in PCF can take effect and become saturable at a lower test-power level; As a result, the cell-type SA has a lower I_sat_. Besides, considering that the thickness of WS_2_ nanosheets is nonuniform, the stronger light-matter interaction can excite the thicker nanosheets to take effect, which might enhance the amplitude of Δα.

Figure 5 shows the schematic of mode-locked fiber laser with our WS_2_ SA device. The pump source is a laser diode (LD) with emission centered at 975 nm. A piece of 2.4 m EDF is used as the laser gain medium with absorption coefficient of 25 dB/m@980 nm (IsoGain^TM^ I-25(980/125), Fibercore). The pump is delivered into EDF via a wavelength division multiplexer (WDM) coupler. An isolator (ISO) is used to ensure unidirectional operation. A fused fiber OC is used to extract 30% energy from the cavity. The cell-type WS_2_ SA is inserted between the three-spool polarization controller (PC) and the OC. Apart from the gain fiber, the remained fiber in cavity is SMF-28 fiber. The total cavity length is around 10.57 m. Assuming that the group velocity dispersion of SMF-28 fiber and the EDF fiber at 1560 nm are ~−23.9 ps^2^/km and 40 ps^2^/km, respectively, the net cavity dispersion is estimated to be −0.099 ps^2^. The laser performance is observed using an optical spectrum analyzer (Yokogawa, AQ6370B), 1 GHz digital oscilloscope (Tektronix, DPO7104C), 3 GHz RF spectrum analyzer (Agilent, N9320A) coupled with a 15 GHz photodetector (EOT, ET-3500FEXT), and an optical autocorrelator (APE, PulseCheck).

Mode-locked operation is initiated at pump power of 37 mW and stabilized 41 mW. Figure 6(a) shows the typical spectrum of mode-locked pulses. The generated optical soliton is centered at 1563.8 nm with a 3-dB bandwidth of 5.19 nm. Figure 6(b) shows the autocorrelation trace, which has a full width at half maximum width (T_FWHM_) of 808 fs. Consequently, the pulse duration *τ* is around 524 fs if a sech^2^ pulse profile is assumed. Thus, the time-bandwidth product (TBP) is 0.333, indicating that the output pulse is slightly chirped. The radio frequency (RF) spectra of the laser are shown Fig. 6(c,d). The fundamental repetition frequency is 19.57 MHz. The electrical signal-to-noise ratio (SNR) is 60.5 dB measured with a 1 kHz resolution bandwidth (RBW). Further increasing the pump power to 102 mW, the pulse fission is observed. The average output power at fundamental frequency repetition was 2.64 mW, corresponding to single pulse energy of 133.6 pJ.

In conclusion, we have developed a PCF-based cell-type SA by filling the SMPCF with WS_2_ nanosheets. It proves that this cell-type SA has the inherent merits, such as: 1) Low IL by shortening the PCF segment; 2) Suppression of the guiding mode distortion effect by using a SMPCF. Therefore, the cell-type SA design can overcome the limits of application for PCF-based SA. The modulation depth, saturable intensity, and non-saturable loss of this SA are measured to be 3.53%, 159 MW/cm^2^ and 23.2%, respectively. Based on this SA, a passively mode-locked EDF laser has been achieved with pulse duration of 808 fs and repetition rate of 19.57 MHz, and signal-noise-ratio (SNR) of 60.5 dB. Our results demonstrate that the cell-type WS_2_ nanosheets SA can serve as a good candidate for short-pulse mode locker.

## Additional Information

**How to cite this article**: Yan, P. *et al.* Passively mode-locked fiber laser by a cell-type WS_2_ nanosheets saturable absorber. *Sci. Rep.* **5**, 12587; doi: 10.1038/srep12587 (2015).

## Figures and Tables

**Figure 1:**
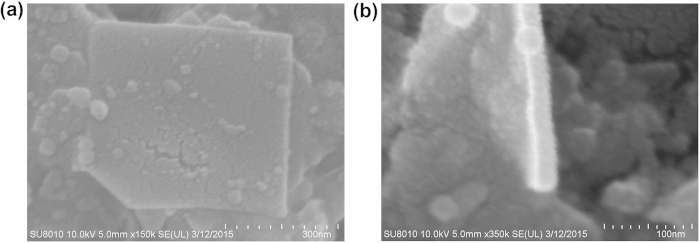
SEM of surface and thickness of WS_2_ nanosheet.

**Figure 2:**
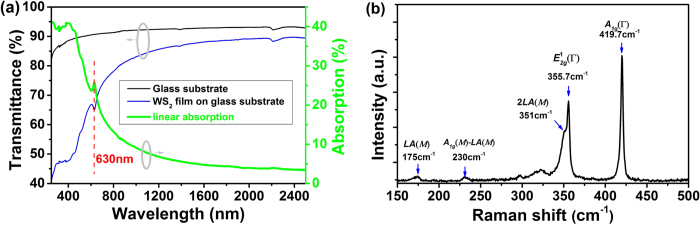
Linear-absorption spectrum and Raman spectrum of WS_2_ nanosheets on silica glass.

**Figure 3:**
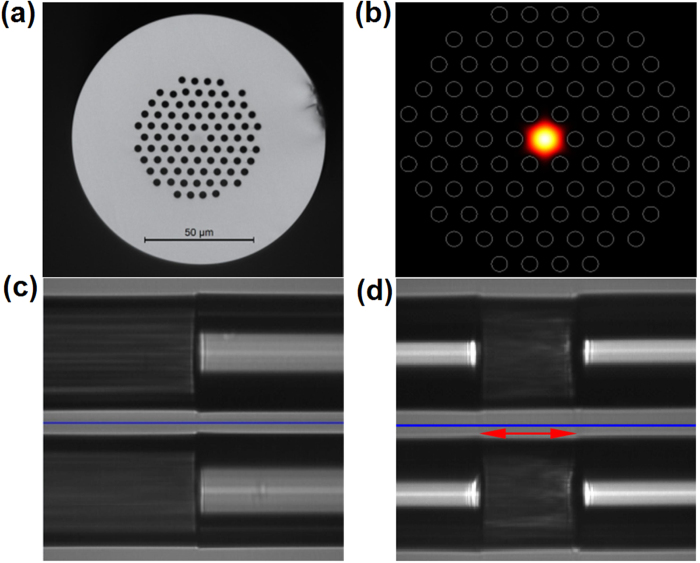
(**a**,**b**) PCF cross section and calculated mode distribution; (**c**,**d**) Splicing point and cell-type SA device.

**Figure 4:**
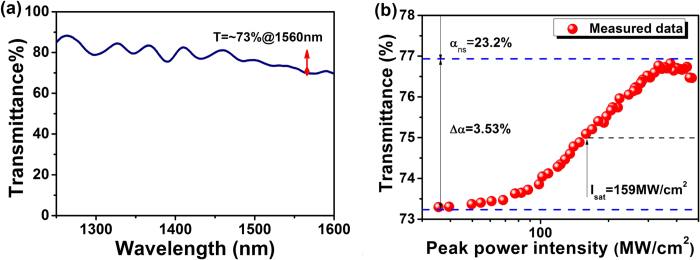
(**a**) Linear transmittance measured in the spectral spanning from 1250 nm to 1600 nm; (**b**) Nonlinear transmittance with Δα of 3.53%, Isat of 159 MW/cm^2^, and α_ns_ of 23.2%.

**Figure 5:**
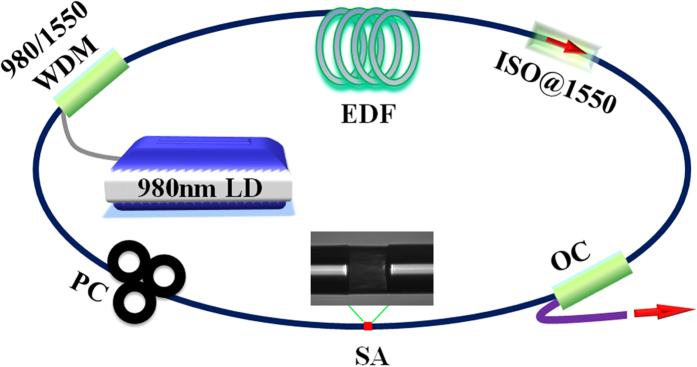
Schematic of mode-locked fiber laser based on cell-type WS_2_ SA.

**Figure 6:**
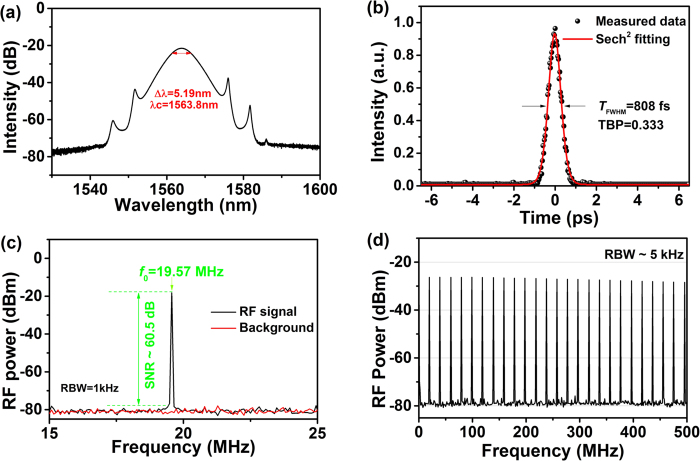
Laser performance. (**a**) Optical spectrum; (**b**) Autocorrelation trace; (**c**,**d**) RF spectra.
